# Impact of meltwater flow intensity on the spatiotemporal heterogeneity of microbial mats in the McMurdo Dry Valleys, Antarctica

**DOI:** 10.1038/s43705-022-00202-8

**Published:** 2023-01-23

**Authors:** A. Zoumplis, B. Kolody, D. Kaul, H. Zheng, P. Venepally, D. M. McKnight, C. Takacs-Vesbach, A. DeVries, A. E. Allen

**Affiliations:** 1 Scripps Institution of Oceanography, University of California, San Diego, CA USA; 2 Microbial and Environmental Genomics Group, J. Craig Venter Institute, La Jolla, CA USA; 3 Earth and Planetary Science, University of California, Berkeley, Berkeley, CA USA; 4 Institute of Arctic and Alpine Research, University of Colorado, Boulder, CO USA; 5 Department of Biology, University of New Mexico, Albuquerque, NM USA; 6 Evolution, Ecology and Behavior, University of Illinois at Urbana-Champaign, Urbana, IL USA

**Keywords:** Molecular ecology, Microbial ecology

## Abstract

The meltwater streams of the McMurdo Dry Valleys are hot spots of biological diversity in the climate-sensitive polar desert landscape. Microbial mats, largely comprised of cyanobacteria, dominate the streams which flow for a brief window of time (~10 weeks) over the austral summer. These communities, critical to nutrient and carbon cycling, display previously uncharacterized patterns of rapid destabilization and recovery upon exposure to variable and physiologically detrimental conditions. Here, we characterize changes in biodiversity, transcriptional responses and activity of microbial mats in response to hydrological disturbance over spatiotemporal gradients. While diverse metabolic strategies persist between marginal mats and main channel mats, data collected from 4 time points during the austral summer revealed a homogenization of the mat communities during the mid-season peak meltwater flow, directly influencing the biogeochemical roles of this stream ecosystem. Gene expression pattern analyses identified strong functional sensitivities of nitrogen-fixing marginal mats to changes in hydrological activities. Stress response markers detailed the environmental challenges of each microhabitat and the molecular mechanisms underpinning survival in a polar desert ecosystem at the forefront of climate change. At mid and end points in the flow cycle, mobile genetic elements were upregulated across all mat types indicating high degrees of genome evolvability and transcriptional synchronies. Additionally, we identified novel antifreeze activity in the stream microbial mats indicating the presence of ice-binding proteins (IBPs). Cumulatively, these data provide a new view of active intra-stream diversity, biotic interactions and alterations in ecosystem function over a high-flow hydrological regime.

## Introduction

The McMurdo Dry Valleys (MDVs) are considered one of the harshest environments on Earth [1]. Low temperatures, rapid freeze/thaw cycles, aridity, variations in light regimes, steep chemical and salt gradients, and nutrient bioavailability all pose challenges to life in this evolving landscape [2–7]. In the early 1990’s the MDVs experienced a decadal cooling trend [8]. In 2006 this cooling trend terminated and the MDVs have since experienced higher and more variable temperatures with high-flow and flood events occurring more frequently [9, 10]. Warming events have resulted in rapid and sustained effects on microbial communities in the polar desert landscape [11–13]. Data presented in this study on the stability of keystone species and processes in response to natural, hydrological variation sheds light on the future predictions of ecosystem stability in response to climate-driven variation in the Antarctic Dry Valleys.

Microbes dominate several ecosystems within the polar desert including the glaciers, soils, ponds, meltwater streams, and perennial ice covered lakes [14–19]. The glacial meltwater streams constitute the most biodiverse habitat of the MDVs [19–21]. Throughout the austral summer, rising temperatures and increasing solar radiation cause glacial melting that flows downwards, saturating stream beds and underlying hyporheic zones [22]. Streams flow is characterized by daily pulses driven by changing sun angles and punctuated periods of high flow for 2 to 3 months before returning to a desiccated, frozen state over the austral winter [23].

Most of the biomass in the MDVs is in the form of benthic microbial mats [24]. Cyanobacteria form the basis of these cohesive mats which include bacteria, eukaryotic algae, protists and micro-invertebrates [24, 25]. Organisms within the mats are thought to be highly adapted to life in a polar desert. Adaptations such as rapid metabolomic and reproductive reactivation, efficient photosystems, and nutrient scavenging protect against intermittent water activity, low light levels and oligotrophy, respectively [5, 26, 27]. Stream microbial mats typically form along stream margins and within main channels [28]. Flow dynamics of the streams create hydration gradients between these locations establishing niches of microbial communities [17, 28]. Within an individual stream, the pigment diversity associated with stream mats of differing habitats is a potential indication of selective pressure related to solar irradiance [29]. Orange mats are predominately found in the main stream channel forming cohesive benthic mats [30]. Green mats, typically, are found in the main stream channel attached to rocks, with black mats forming near the stream margin [28]. The observed red mats are variably located intermediately between margin and channel habitats. The presence and biomass of these mats have been historically monitored based on photosynthetic pigments [17, 21, 30, 31].

Placement of a mat within a stream may affect the degree of light refraction, availability of nutrients, exposure to low temperatures and periodic desiccation and other factors that may drive community structure [31]. Furthermore, these hydrological and environmental conditions are stream dependent and change throughout the austral summer with peak flow occurring during the month of January [31]. Past studies have emphasized flow dynamics and hydrological regimes as important drivers of stream bacteria and diatom community structures [6, 17, 21, 32, 33]. Limitations of biodiversity studies conducted in the meltwater streams include classifications based on morphological characterizations which may underestimate the number of species within communities. Likewise, DNA methods may be placing heavy emphasis on nonfunctioning organisms and potentially inactive genetic material. Characterizing the active communities of the meltwater streams to in situ flow cycles is critical to understanding the stability of this ecosystem in a climate-sensitive region.

To date, there has not been an in depth look at the active biodiversity and transcriptional response of in situ meltwater stream communities to hydrological regimes over ecologically meaningful spatial and temporal scales. Here, we characterize within stream mat functional variation over an austral season and establish the molecular underpinnings of survival in this unique and changing environment. Our results provide a new view into the critical roles of microbial mats in the biological uptake and biogeochemical cycling of nutrients in a rapidly changing, climate-sensitive environment. The patterns identified in this study can provide insights into microbial community responses to future climate-driven hydrological changes and flood events in a warming polar ecosystem. Furthermore, results identify genes and pathways with potential novel biotechnological application.

## Materials and methods

### Sample collection

**Fig. 1:**
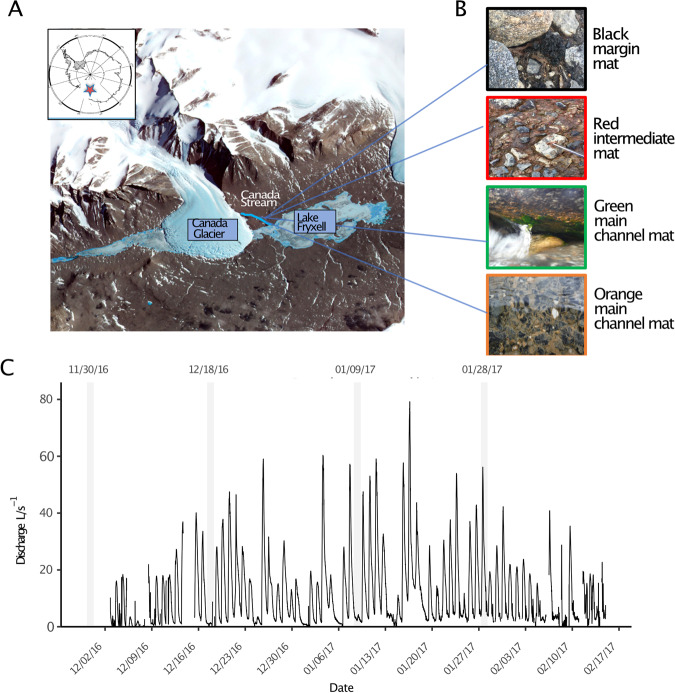
Sampling site and stream features. **A** Map of Lake Fryxell Basin, Taylor Valley, Antarctica showing placement of the Canada meltwater stream. **B** Visuals of the four mat types found along the Canada stream margins and within the main channel. **C** Hydrograph showing the seasonal discharge rate (L/s) of Canada stream flow from data collected every 15 minutes over 12 weeks.

Algal mat samples were collected during the 2016–2017 field season from a pre-existing microbial sampling transect established by the McMurdo Long Term Ecological Research (MCM-LTER) program [28]. Sampling was conducted at the Canada Stream transect (−77.6132414, 163.0526875) within the Taylor Valley of the McMurdo Dry Valleys (Fig. 1A). The hydrological record from the Canada Stream has been maintained consistently with annual flow data measurements available since 1990. Canada Stream is the most consistently flowing stream in the Fryxell basin receiving flow from the East facing tongue of the Canada Glacier that drains into two ponds prior to entering a well-defined channel [22]. Flow in Canada Stream begins earlier and ends later in the season than other Fryxell basin streams and has a greater probability of high flows (100 L/s) which can result in the scouring of microbial mats [22, 34]. On average Canada stream has lower values of total N (1.83 μM), soluble reactive phosphorous (0.13 μM), dissolved organic carbon (0.031 mM) and total dissolved solids (9.03 mg l^−1^) in comparison to other McMurdo Dry Valleys streams [21].

Microbial mats were collected from the Canada Stream margin, channel and intermediate sites at four time points throughout the summer season to capture flow dynamics that are hypothesized to drive microhabitat diversity. Samples were collected around same time of day (+/− an hour) to mitigate circadian influence. Red and black mats were collected near and on the stream margin respectively (Fig. 1B). Orange and green mats were collected from the main channel. Only black and red mats were visibly present at the early season sampling time point (November 2016). Orange and green main channel mats were indistinguishable from sediment prior to more consistent stream flow occurring later in the season. Mat samples for transcriptomics and rRNA analyses were collected in triplicates using an EtOH-sterilized #13 brass cork borer (227 mm^2^) and forceps, and transferred into sterile 2 ml cryovials and covered with ~1 ml of RNALater. Cryovials were then flash frozen in liquid nitrogen and stored at −80 °C. Mat samples collected for the purpose of antifreeze activity assays were collected in sterile whirlpack bags and stored at −20 °C.

### Methods

RNA and DNA were extracted from frozen, RNAlater^TM^ (Thermo Fisher Scientific) preserved microbial mat samples in accordance with NucleoMag RNA (Macherey-Nagel) and NucleoMag Plant (Macherey-Nagel) kit protocols. The V4-V5 region and V4 region of the 16S and 18S ribosomal RNA (rRNA) was amplified, independently from RNA and DNA using universal primers [35–37]. Full methods for sample processing for sequencing are provided in Supplementary File 1. Libraries were sequenced using the Illumina MiSeq platform (2 × 300 bp) generating a total of ~2 M reads with an average number of 150,000 reads per sample for both 16S and 18S datasets. Sequences were quality filtered and amplicon sequence variants (ASVs) were generated using the DADA2 [38] module in QIIME2 (version 2019.4) [39] with default thresholds for expected error and the following length truncation parameters: 250 bp for forward truncation (--p-trunc-len-f) and 200 bp for reverse truncation (--p-trunc-len-r).

Taxonomic annotations for 16S and 18S rRNA ASVs were assigned through rRNA reference databases including SILVA (version 132) and NCBI-NT/NR (Date of access 2017-11-29), respectively. Plastids were filtered out using QIIME’s taxonomy-based filtering with “qiime taxa filter-table” and “qiime taxa filter-seqs” to exclude features (ASVs) with taxa corresponding to mitochondria and chloroplast. Alpha and beta diversity metrics were calculated using the “core-metrics-phylogenetic” method using default parameters and visualized using ‘Emperor’ tool in QIIME 2 (version 2019.4).

Using extracted RNA as input, ribosomal RNA was removed using Ribo-Zero Magnetic kits (Illumina). Metatranscriptomes were then generated from the isolated mRNA. cDNA synthesis and simultaneous amplification of polyA as well as total RNA results in substantial coverage of prokaryotic and eukaryotic mRNA [40, 41]. Metatranscriptomic libraries were sequenced on the Illumina HiSeq 4000 platform generating approximately 100 Gb/400 million reads per lane, ~5 Gb/10 million paired reads per sample. Post-sequencing, reads were filtered and trimmed for removal of primers, adapters, and low quality sequences. Ribopicker (version 0.4.3) [42] was used to identify and remove rRNA. Transcripts were assembled into contigs with CLC Genomics (version) and open reading frames (ORFs) were predicted with FragGeneScan (version 1.31) [43]. ORFs were annotated de novo for function against the Kyoto Encyclopedia of Genes and Genomes (KEGG) [44], Pfam [45] and hidden Markov model (HMM) searches [46], and the in-house comprehensive reference database, PhyloDB (version 1.076) for taxonomic annotation of these ORF sequences.

For differential expression analysis, between mat type, triplicate samples collected on 12/18/16, 1/9/17 and 1/28/17 were pooled based on mat type. Pairwise differential expression analyses of transcripts between mat types were performed using the R package edgeR (version 3.10.5) [47]. Counts were normalized using the “calcNormFactors” function and an extract test with tagwise dispersion was used to test for differential expression across mat types. Resulting *p* values were False Discovery Rate (FDR) corrected for multiple testing (Benjamini-Hochberg) and FDR < 0.05 was used as a significance threshold.

In order to more comprehensively annotate ORF function, ORFs were grouped into clusters of similar amino acid sequences using the Markov Cluster Algorithm (MCL). Directional edge weights were set as the ratio of pairwise- to self- BLASTP scores, and default parameters were used to assign ORFs to clusters. A consensus cluster annotation was called by clusters being statistically enriched in annotations with a Fisher’s exact test (*p* < 0.05). Clusters that were annotated as having the same function were grouped into “functional clusters.” A weighted gene co-expression network analysis (WGCNA) (version 1.70.3) [48] was performed as previously described [49] to identify groups, or modules, of functional clusters with synchronous expression patterns across the four mat types. Only functional clusters with at least 500 reads in 80% of samples were included in the WGCNA analysis. WGCNA was run on library-normalized counts. Modules were detected using the blockwiseModules function using a power function exponent, b, of 14 to optimize scale-free topology, a minimum module size of 30 clusters, and parameters detectCutHeight = 0.995, reassignThreshold = 0, mergeCutHeight = 0.5. The top 5 most abundant clusters in each module were plotted using igraph and tcltk.

Orange, green, red and black mat samples were collected from the Canada stream and stored and shipped at −20 °C for antifreeze activity assays. One gram of each mat type was added to a 2 ml microcentrifuge tube filled with 200 μl) distilled water. The mats were then put through 5 freeze thaw cycles (−20 °C for 30 min followed by a 10 min thaw at room temperature). Samples were spun down for 10 min at 5000 rpm at 4 °C and the supernatant was collected.

A Clifton Nanoliter Cryoscope (Clifton, New Jersey) was used to determine the freezing and melting points of the lysed mat samples. The cryoscope is fitted with a cold stage utilizing a temperature-controlled Peltier sample holder. Temperatures are accurate to 0.01 °C. Each of the 600 μm diameter sample holder wells were loaded with heavy microscope immersion oil (Type B). Ten nanoliters of the collected microbial mat supernatant was inserted into the center of each oil-filled well with a micropipette. Controls were distilled water (Control 1) and 1000 mOsmol standard (Control 2).

Once loaded, samples were cooled to −40 °C. The frozen wells were warmed until only a single isolated 10um ice crystal was left in the well. Melting points of the isolated crystal were determined by lowing and raising the temperatures until the melting velocity was undetectable. The melting points of solutions with no antifreeze protein activity were at equilibrium with the freezing point. Single ice crystals were slowly cooled at a rate of 0.074 °C/min. The non-equilibrium freezing points were recorded and the difference from the equilibrium melting point is reported as the thermal hysteresis. In additional experiments, the single, small ice crystal underwent a 30 min period of annealing at a temperature slightly below the melting point before cooling after which the thermal hysteresis was recorded.

## Results

### Sensitivity of community structuring to meltwater influx

**Fig. 2:**
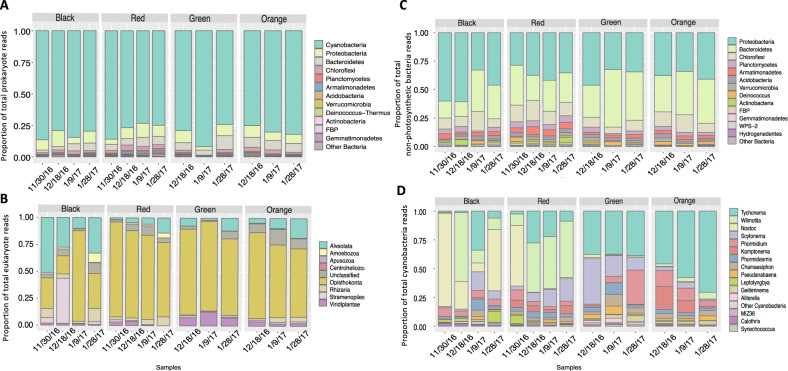
Microbial mat community structuring. Relative abundance of (**A**) prokaryotic, (**B**) eukaryotic, (**C**) non-cyanobacterial, (**D**) cyanobacterial community members from non-plastid 16S and 18S rRNA ASVs.

Ash free dry mass (AFDM) and chlorophyll *a* analyses were conducted on mat samples collected early January. AFDM values were significantly differentiated among black/red mats vs. green/orange mats (Table S1). Black mats reported the highest ratio of AFDM to chl *a*, followed by red mats (Table S1). This result is likely due to elevated detritus deposition to the stream marginal mats as well as higher proportions of non-chlorophyll possessing organisms (Fig. 2B). Eukaryotic species richness was highest in orange and early season black mats (Fig. S1). Sharp drop-offs in observed species for eukaryotes occurred in black and red mat as the season progressed (Fig. S2). Prokaryotic species richness remained relatively consistent throughout the season across mat types (Figs. S3, S4).

**Fig. 3:**
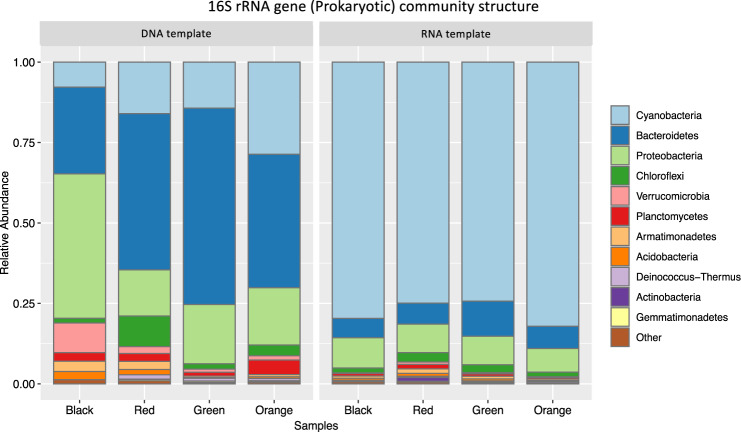
Template dependent community structuring. Comparison of prokaryote relative abundances using single data points collected from 01/28/17 black, red, green and orange mats showing differences between 16S rDNA community sequenced from DNA vs RNA template.

Across 14 samples, 15,477 ASVs were generated with 8611 prokaryotic, 16S (V4-V5 region) and 6,866 eukaryotic, 18S (V4 region) sequences. Prokaryotic 16S rRNA communities across all mat types were dominated by Cyanobacteria (80%). Other major groups of bacteria included Proteobacteria (8%) Bacteroidetes (6%), Chloroflexi (2%) and Planctomycetes (0.80%) (Fig. 2A). The dominance of Cyanobacteria over the non-phototrophic sequences in the prokaryotic community is in contrast to the relatively low Cyanobacteria abundances (~35%) observed in other similar microbial mat community studies in polar, coastal and freshwater environments (Fig. 3) [21, 27, 50, 51]. We attribute these differences to DNA vs. RNA sequencing where more of the active community is revealed (Fig. 3). RNA sequencing is preferred for determining the viable community over DNA and morphological methods given its rapid degradation in non-viable cells [52]. Pre-plastid filtering, the green mats contained a higher proportion of chloroplasts in the DNA community (30%) vs. the RNA community (6%). Chloroplast based sequences were minimally present in red, black and orange mats (<0.3% of cyanobacteria ASVs).

Of the eukaryotes, Opisthokonta averaged a high proportion (66%) of the 18S rRNA community across all mats types, followed by Alveolata (14%), Stramenopiles (4%), Rhizaria (4%), Viridiplantae (4%) and Amoebozoa (1%) (Fig. 2B). A less dominant group, Apusozoa (0.05%), displayed a limited presence in black and orange mats only. A more detailed look into the dominant Opisthokonta reveals taxa group variation among mat types. Rotifera averaged over half of the metazoan community for orange and red mats, whereas green mats are dominated by Tardigrada. Black mats have the highest proportion of Nematoda of the mat types (Fig. S5).

Additional sampling was conducted throughout the season in order to detect community changes over the austral summer. The eukaryotic communities of orange, green and red mats remained relatively stable over time, while black mats on the stream margin experienced strong shifts in taxa groups in both the prokaryotic and eukaryotic communities (Fig. 2). Early season black mats show peaks in Alveolata and Stramenopile, while Opisthokonta dominate the mid-season sample. Late season black mats show a decline in Opisthokonta and an increase in Amoebozoa and Rhizaria. Mid-season black mats, where stream flow is at its maximum, resemble the taxa structure of the green and orange stream bed mats. This shift is evident across multiple taxa groups, particularly in the dominant cyanobacteria communities where black mats experience a mid-season shift from *Nostoc* and *Wilmottia* to *Tychonema* and *Scytonema* before a reversion in late season (Fig. 2).

**Fig. 4:**
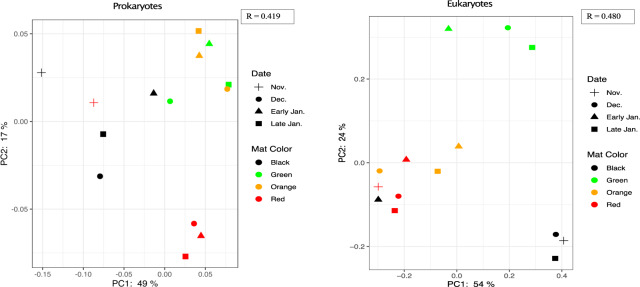
Principal coordinate analyses (PCoA). UniFrac weighted distance matrices with ANOSIM values generated from (left)16S rRNA and (right) 18S rRNA ASVs. Shapes of plotted points denote sample collection date and colors denote mat type of origin.

Principle coordinate analysis (PCoA) was performed on prokaryotic (16S) and eukaryotic (18S) rRNA communities using weighted unifrac distance metric. The PCoA figure visualizes single replicates of the mat types collected over the various sampling time points (Fig. 4). Significant clustering was found in both 16S and 18S rRNA data (*p* value < 0.01, ANOSIM R-statistic 0.307 and 0.480 respectively) (Fig. 4). Beta diversity clustering in prokaryotes reflected a similar community structure as observed in the taxonomic analyses. November, early season, red and black mat types cluster together. Though displaying distinct community signatures, mid-season mats of all types generally cluster together. Red mats appear to have the most dissimilar prokaryotic communities among the four mat types, largely dominated by the cyanobacteria, *Wilmottia* (Figs. 2C, 4). Eukaryotic clustering patterns show clustering between black, red and orange mats with an isolated green mat cluster (Fig. 4). Clustering was strongest among the Rhizaria and Metazoa groups indicating higher similarities within samples of a particular mat type and higher dissimilarities between the different mat types (*p* value <0.01, ANOSIM R-statistic 0.525 and 0.456 respectively). Regardless of temporal distance, eukaryotes were grouped by mat type indicating a lesser impact of hydrological drivers on eukaryotic community structuring (Fig. 4).

The black marginal mats and red, intermediate mats were the most susceptible to shifts in prokaryotic taxa groups over time with channel mat taxa remaining stable. These shifts occurred over a time period of weeks indicating rapid community turnover with changes in flow patterns. Marginal mats are subject to scouring during high-flow periods. Particulate organic matter (POM) sampled over diel flow cycles showed an increase in POM concentration with increased water levels [34]. During these daily flow pulses, shear forces act on the microbial mats [34]. The most predominate form of POM in transport through the streams and hyporheic zones is *Nostoc* derived, as determined by the isotopic signature of N-fixation [53]. N-fixation within microbial mats primarily occurs by *Nostoc* [54].These results support our findings of unstable marginal mat communities. A reduction in flow near the end of the austral summer season resulted in the recovery of *Nostoc* that dominated the marginal mats at the beginning of the sampling period. The rapid recovery of this community to its pre-flow state indicates resilience of these critical, keystone microbes.

Past studies have sampled mat communities across several streams in the Miers, Taylor and Wright Valleys to detect patterns in prokaryotic diversity [21]. Though phenotypical attributes including pigmentation and structural layering and the spatial distribution of these mats appear similar among different streams, there is significant variation in seasonal flow conditions [33]. Ultimately, these intra-stream processes and physical controls on diversity could explain the lack of homogeneity among meltwater stream mats and potentially why a black, marginal mat from a stream at peak flow may appear similar in community structure to orange, channel mats at other locations, among other observed discrepancies.

### Nutrient cycling and stress responses are significantly differentiated by mat type

**Fig. 5:**
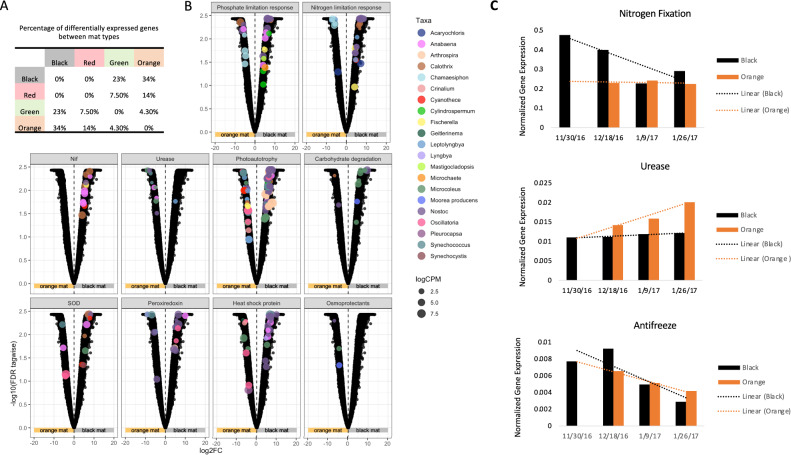
Differential Expression Analyses. **A** Venn diagram showing the percentage of significantly differentially expressed ORFs between mat types. **B** Differential expression of ORFs between orange and black mat pseudo replicates (12/18/16, 1/9/17 and 1/28/17). The following facet labels represent the collection of pathway-critical genes, referenced in Chan et al. [85] to depict functional processes: Nutrient limitation: “Phosphate limitation response”, “Nitrogen limitation response”. The following genes were used for detection of phosphate limitation: Alkaline phosphatase response regulators, high affinity phosphate-specific ABC transport genes, phoA, phoB, pstSCAB. For nitrogen limitation the following genes were used: glutamine synthase and regulators glnA, glnR, tnrA. Nitrogen cycling: Nitrogen fixation “Nif”, “Urease”. Carbon cycling “Photoautotrophy”, “Carbohydrate degradation”. Stress responses: Superoxide Dismutase “SOD”, “Peroxiredoxin”, “Heat shock protein” and “Osmoprotectants”. Significantly differentially expressed ORFs are colored by taxonomy and scaled by abundance. **C** Library-normalized expression of (top) nitrogen fixation, (middle) urease, and (bottom) antifreeze ORFs from black and orange mats over time. This analysis highlights the expression of several individual genes over at the single time point level. Additionally no orange mats were present in the streams at the November time point.

Across all mat types, orange and black mats had the highest percentage of significantly differentially expressed genes (34%) followed by green and black mats (23%) (Fig. 5A). Furthermore, black and red mats had no significantly differentially expressed genes (Fig. 5A). The transcriptional profiles of the orange and black mats showed uniquely enriched genes corresponding to geobiological processes and stress responses (Fig. 5B). This analysis utilizes pseudo replicates of mat type due to the variation in time points but this shows differential expression between mat types collected during the 2016/2017 austral summer season. Identification of these functional disparities broadens our understanding of the structuring of these communities and the degree of physiological plasticity required for survival in this transient environment.

#### Nitrogen cycling

Diazotrophs and their role in the Dry Valleys microbial communities have long been documented through numerous diversity and nitrogen (N) cycling activity studies [53–55]. N-fixing cyanobacteria are known to be a primary source of N to meltwater streams along with glacier-derived input [56]. Black marginal mats in particular are thought to be an important niche for generating bioavailable N for the biosphere. Differential gene expression analysis between black and orange mat types provides new insights into the N cycling strategies between these communities. Black mats show upregulation of nitrogenase genes in several species of cyanobacteria including *Anabena*, *Calothrix*, and *Nostoc* (Fig. 5B). In regard to N-fixing capabilities of *Nostoc*-dominated black mats, our data show influence of these mats on N cycling varies over the season as nitrogen fixation genes are upregulated during early and late low-flow time points while expression declines during mid-season peak flow (Fig. 5C).

Orange mats displayed an abundance of urease genes indicating catabolism of urea into NH_4_ and CO_2_ (Fig. 5B)_._ The ability to utilize and repurpose organic N is likely an important adaptation in this N-limited environment. In the meltwater streams, urea is produced by micro-invertebrates. Our findings suggest that *Anabena*, *Leptolyngbya*, *Microcoleus*, *Nostoc* and *Synechococcus* are key taxa for urea utilization (Fig. 5B). Orange mat expression of the urease gene indicative of organic N (urea) utilization increased from early season to late season (Fig. 5C).

Urea, NH_4,_ NO^2^ and NO^3^ transport genes along with NO^2^ and NO^3^ reductases were all present in our dataset. Our functional analysis did not detect any genes related to nitrification in any of the four mats types. Potential for nitrification and the accumulation of N has primarily been found to occur in the stream sediment and hyporheic zone [57, 58].

#### Carbon cycling

The identification of carbon fixation related enzymes revealed an abundance of autotrophic organisms in the mats. A search for alternate carbon source utilization yielded support for the catabolic degradation of starches, cellulose, and hemicellulose among cyanobacteria. The use of exogenous carbon sources is not surprising in cyanobacteria evolved in ecosystems undergoing natural, extended periods of darkness. In this dataset, we identify several genera of cyanobacteria potentially capable of heterotrophic metabolism including *Leptolyngbya*, *Microcoleus*, *Nostoc, Moorea, and Calothrix* (Fig. 5B).

#### Stress responses

Low temperatures and freeze events have been shown to upregulate stress responses such as lipid biosynthesis, fatty acid desaturases and heat shock proteins (HSP), in cold dwelling microorganisms [59, 60]. The biosynthesis and transport of compatible solutes including glycine betaine, ectoine and trehalose prevent water loss from cells as ice forms [61]. Here, we find marginal mats primarily undergoing temperature stress as indicated by upregulation of HSP family genes (Fig. 5B). At the beginning and end of the austral summer season, black mats are periodically exposed to air temperatures during periods of low flow. Air temperatures in the Fryxell basin averaged −2.92 °C (σ 3.16) and −1.45 °C (σ 2.22) throughout the December and January months, respectively. These temperatures are significantly lower than the average water temperatures recorded by the Canada stream gauges for December 4.08 °C (σ 2.14) and January 3.48 °C (σ 2.51). The initiation and termination of stream flow, occurring early in season, can result in freeze/thaw cycles that potentially explain the upregulation of genes encoding heat shock proteins seen in our data (Fig. 5B). Additionally, desiccation and oxidative stress responses have also been identified in marginal mats (Fig. 5B). In contrast, channel mat organisms expressed elevated osmoprotectant transcripts (Fig. 5B). Cyanobacteria species from both mat types appear to be nitrogen and phosphorous limited though black mats exhibit a more taxonomically diverse response to these nutrient deficiencies (Fig. 5B).

This differential expression analysis enabled the discernment of specific contributions of mat types to biogeochemical processes, including nitrogen and carbon cycling, and identified stress responses providing insight into environmental challenges. Observing gene expression over the austral summer season provided insights into the abruptness and destabilization of critical functions (i.e., nitrogen fixation) in lotic periods. While our data on gene expression does not represent a direct measure of biogeochemical processes, it is indicative of particular biogeochemical transformations. Also, while our data robustly illustrates relative level of metabolic activity, we are restricted by lack of absolute quantitation; therefore, we are somewhat limited to observations regarding the prevalence and relative importance biogeochemical patterns and processes rather than specifically quantified fluxes.

### Weighted correlation network analysis reveals hydrological drivers of functional synchronies

**Fig. 6:**
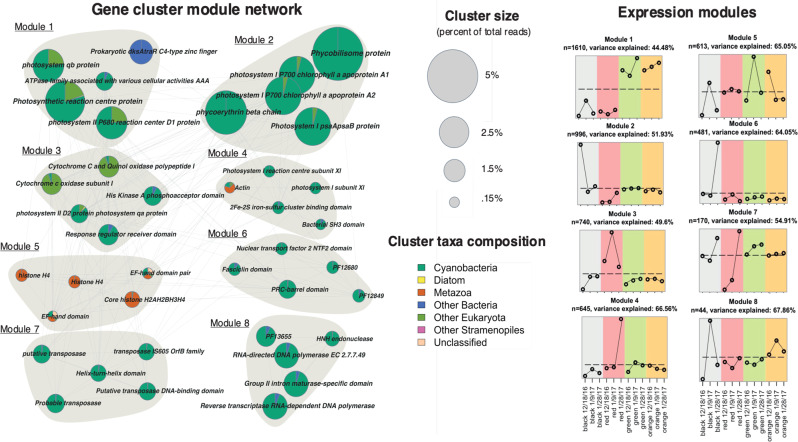
Visualization of networks of co-expression modules as determined by WGCNA. Pie charts represent highly abundant functional clusters of ORFS colored by taxonomic contribution and sized by percent of total reads (*left*). Co-expression patterns of modules colored by mat type (*right*). Subtitle describes number (n) of ORFs in each module as well as percentage of variance explained by each module’s expression profile.

The weighted correlation network analysis (WGCNA) identified eight modules (Fig. 6) of co-expressed functional clusters. A majority of these clusters fall into modules that have expression patterns that appear to be responding to mat type and flow dynamics (Fig. 6). The number of clusters in each module ranges from 1610 (module 1) to 44 (module 8) (Fig. 6). Module 1 displays a clear difference in activity established by the upregulation in green and orange mats compared to the black and red mats (Fig. 6). Top gene activities of modules 1, 3, 5 appear to be driven by high stream flow (Fig. 6). Functions exhibiting peaks in hydration periods include photosystem II, eukaryote related cytochrome c oxidase and histone ORFs (Fig. 6). Modules 2, 4, 6 and 7 are correlated to low-flow regimes where top functions include photosystem I, binding proteins including fasciclin and PF12849 (periplasmic binding protein domain), and PF12680 (antibiotic formation) (Fig. 6). Likely, photoinhibition of the PSII is caused by exposure to higher irradiance levels and accumulation of reactive oxygen species (ROS) in the low-flow modules whereas we see higher expression of the protected PSI [62, 63]. The ancient fasciclin domain is present across many taxa groups and is important in cell interaction with the external environment [64]. As a significant component in the extracellular matrix, fasciclin plays a variety of roles in cell adhesion and stress management [64–66]. Here, we see elevated transcripts of cyanobacterial fasciclin proteins in black mats at the termination of stream flow. (Fig. 6). Module 7 is comprised largely of transposases that are upregulated across all mat types at the end of flow indicating a potential stress response to the start of the desiccation period (Fig. 6). Orange and black mats both exhibit an upregulation of genes encoded by viruses during periods of high-flow apparent in module 8 (Fig. 6).

Functionally, black marginal mats behaved more similarly to red intermediate mats than to distal channel mats. The black, marginal mats appear to be the most transcriptionally sensitive to hydrological activity within the stream. External environment triggering occurs throughout the austral summer season in these mats as visualized by mat-type specific upregulation of gene clusters in response to changes in flow over time. Modules 2 and 6 display an upregulation in the function of marginal mats during low-flow intervals (Fig. 6). Conversely, gene expression patterns in the black, marginal mats appears to synchronize with other mat types over peak flow in modules 5 and 8 (Fig. 6). The cluster expression synchronies in module 5 are functionally annotated as metazoan which can in part, be due to a shift in the marginal mat community to metazoan taxa with increased flow (Figs. 2B, 6).

Mobile genetic elements including transposases, homing endonucleases and intron maturases are found to be upregulated in the microbial mats at mid and end points in the flow cycle (Fig. 6). These genes may be part of an adaptive toolkit for the mats resulting in potential trait plasticity in response to the stress of meltwater stream variability. Upregulation of RNA-directed DNA polymerase functional clusters in black and orange mats during peak flow potentially suggest virus mediated alterations of cyanobacterial host physiology or host defenses against viral activity (Fig. 6). Infection-related host phenotypes have not been determined in this study though viral genetic exchange can be advantageous for host evolution.

### Novel thermal hysteresis and antifreeze activity in Dry Valleys microbial mats

**Fig. 7:**
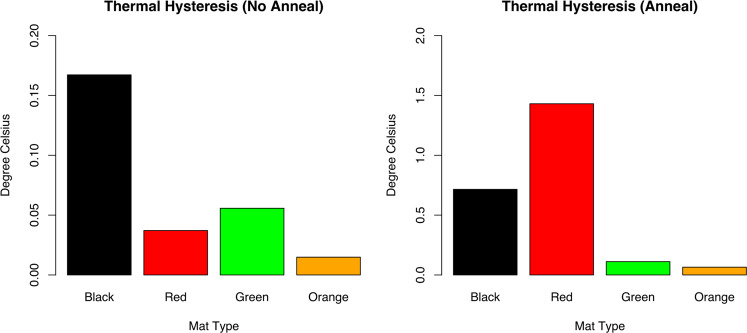
Microbial Mat Antifreeze Activities. Thermal hysteresis antifreeze activities of black, red, green and orange mats with no annealing period (left) and after a thirty minute annealing period (right).

Antifreeze activity assays were conducted as a further analysis of heterogeneity of function in mat types specifically, in regard to freeze/thaw stress responses. All mat types across Canada Stream displayed some degree of thermal hysteresis (TH) in the antifreze assays (Fig. 7). The strongest hysteresis in the ‘no anneal’ time trials was the black mat with a TH of 0.17 °C followed by green mats (0.056 °C), red mats (0.037 °C) and orange mats (0.015 °C). Ice crystal seeds grew as a disc for orange mats which is consistent with controls. Orange mat ice crystal visualization along with thermal hysteresis results indicate little to no antifreeze activity. Black and green mat supernatants displayed faceting on a-axis (prism planes). Red mats displayed growth in patches on basal plane as well as some faceting on a-axis.

An additional antifreeze assay was performed using the same methods previously described with an extra step of a 30 min anneal before cooling the ice crystal. Annealing time along with concentration and slower cooling rates have had significant impacts in increasing the TH activity [67–69]. In this investigation the additional annealing time resulted in a significant increase in TH activity and binding in red (1.4 °C), black (0.72 °C) and green mats (0.11 °C). Black and green mats had relatively high TH variability among samples. Orange mats (0.065 °C) remained at little to no antifreeze activity. Dendritic bursts were evident in black and red mats upon reaching the freezing point threshold (Fig. S7). Green mats displayed evidence of prism plane faceting. These results support the idea that annealing time has a strong influence on the thermal hysteresis activity.

A Hidden Markov Model (HMM) search identified ORFs of the conserved ice-binding-like protein Pfam (PF11999). This Pfam includes the domain of unknown function (DUF3494) which contains ice-binding proteins (IBPs) from a diverse array of organism with varying magnitudes of function [70]. The search yielded a large percentage of prokaryotic derived proteins with several stramenopile and fungi variations. The counts were highest in early season black and red mats in comparison to the lower count orange and green mats (Fig. 5C). The taxonomic representation in the highly expressing black and red mats consists of *Singulisphaera acidiphila* and *Streptomyces sviceus* belonging to the Planctomycetes and Actinobacteria groups, respectively, with the top Eukaryotic hit being the fungi *Zymoseptoria tritici* (Fig. S8). Alternatively, the Planctomycetes species *Pirellula staleyi* and the stramenopile, *Ochromonas* were the most dominant IBP encoding taxa in the orange and green mats (Fig. S8). No taxa described here are exclusive to any one mat.

There is potential for novel structural IBP diversity in the microbial mats as up to 5% of the IBP expressing community in early season black and red mats were from unclassified taxa groups (Fig. S8). Applications of such antifreeze activity described in this analysis expands to food, medical and biotechnology industries. It appears red mats, in particular have moderate antifreeze activity, with a TH reaching ~1 °C [71], under certain freezing conditions. While we have reported a higher TH activity with longer annealing times, additional factors known to increase TH activity include higher IBP concentration and slower freezing rates [71].

## Discussion

The findings in this study detail changes in the active community structures and gene expression patterns of microbial mats in a high-flow meltwater stream. The variability and functional potential of stream communities may play important roles in landscape processes over time. Cyanobacteria, in particular, dominate much larger proportions of microbial mat communities than previously reported in other DNA based studies as determined by our RNA vs DNA diversity analysis. RNA was used as a template for sequencing for the purpose of describing the active community diversity due to it’s quicker degradation in the environmental samples and protein synthesizing potential in comparison to DNA templates [52]. The significant changes to the structure of both prokaryotic and eukaryotic communities in the span of weeks is not uncommon in the polar desert as these microorganisms are known to respond rapidly to environmental changes [72, 73]; However the functional nature and significance of such changes has gone previously undocumented in the vital meltwater ecosystem.

Black, marginal mats contribute heavily to nitrogen fixation and are subsequently responsible for N inputs after depletion of glacier-derived N [53]. These mats, critical to the sustainability of the meltwater ecosystem, undergo destabilization of community structure and function. While we have established some of the mechanistic means of black mat resilience to the harsh, rapidly fluctuating environment, the instability of keystone community members with hydrological variation is striking, representing a distinctive feature of ecosystem function for these streams.

Prior studies on Dry Valleys stream mat diazotrophs indicate the exclusivity of cyanobacteria nifH sequencs (~90% of total sequences) to the *Nostoc* genus [54]. Our taxonomic structuring (Fig. 2) and transcription data (Figs. 5, S9) emphasize the strong influence of *Nostoc* as well as *Calothrix* and *Anabaena* species on the support of this vulnerable, N-limited ecosystem. In the expression of Nitrogenase related ORFs in black mats, Nostocaceae family genera *Nostoc* and *Anabaena* averaged 23% and 18% respectively whereas *Calothrix* averaged 36% across the season (Fig. S9). While functional redundancies are known among the diazotrophs [74], a sharp decline in the dominant Nitrogen-fixing species, *Nostoc* and *Calothrix* mid-season along with a reduction in nitrogenase gene expression indicates a loss of function not compensated for by other mat species. Rather, there is a shift in general N acquisition strategies which transitions from nitrogen fixation to the breakdown of urea via urease during the peak stream flow interval. These distinct functions were largely correlated to mat type. *Anabaena* and *Nostoc* were found to contribute to both the Nitrogen fixation in the black mats and the catabolism of urea in the orange mats. It is unknown the extent of which autotrophic and heterotrophic stream sediment diazotrophs may compensate for the decrease in nitrogenase activity. Sediments contain much higher proportions of heterotrophic diazotroph NifH sequences and are more resistant to short-term disturbances [54, 74].

The distinctions in transcriptional expression among the different mat types establish varying hydrological regimes across relatively small spatiotemporal scales as a driver of functional heterogeneity. Several co-expression modules independently recreate temporal flow patterns measured across the season but exhibit divergences by mat type. These results indicate the potential impact of spatial dynamics and mat specificity on functional diversity. It is to be noted that the data presented in this analysis stems from single time point sampling and variances may not be fully representative of mat transcriptional profiles. Other limitations include the lack of data on Dry Valleys mat circadian cycles. Circadian influence was mitigated by collecting samples around the same time of day (±1 h).

Hydrological variance is noted in several desert ecology studies as the primary driving factor of microbial community relative to temperature, nutrients and other local environmental conditions [21, 33, 75]. Impact of stream flow, temperature and niche filtering has been associated with changes in community structure, and more recent data using functional metabolic pathway prediction analyses is suggestive of overall functional profiles changes in these communities [76]. Here, we establish clear transcriptional disparities occurring with hydrological change in the meltwater stream communities.

Finally, we have presented information on the ice shaping abilities and thermal hysteresis activities on the meltwater stream microbial mats. In particular, black marginal mats and red intermediate mats hosts microbes that have potential uses in food, biotechnology and medical fields. The autecology and cultivability of microbes from these mats is a challenge to the further characterization of taxa specific antifreeze activity. Additional studies on focused on recombinant expression characterization, purification and stability of these potentially novel IBPs could lead to a better understanding of survival through freeze/thaw cycling but also industrial application.

This study provides relevant insight to other desert and stream habitats as functionally important species, such as Nostoc, are prevalent across a wide range of climate-sensitive ecosystems. The polar desert is a region prone to exacerbated effects of the warming climate [77, 78]. Past studies have shown the dramatic and persistent effects on diversity of microbial mat taxa during flood pulse events [11, 12, 79]. With future projections of climate-driven variation [80], it is unknown if these mats will continue to adapt and persist in their polar desert niche.

Moreover, there is a lack of molecular biodiversity and functional expression data for hydrological and solar transition periods in the Dry Valleys meltwater streams. Data on responses of lake bacterioplankton, phytoplankton and protists taxa groups to bimodal solar cycling indicate degrees of metabolic plasticity that should be further explored with modern molecular approaches [81–84]. Likewise, additional replicated stream mat sampling over spatiotemporal gradients and continuous periods can provide a better picture of the biogeochemical contributions and stability of stream biota within the rapidly changing polar desert system.

## Supplementary information

Table S1

Table S2

Figure S1

Figure S2

Figure S3

Figure S4

Figure S5

Figure S6

Figure S7

Figure S8

Figure S9

Supporting Methods

## Data Availability

Raw data has been deposited in the National Center for Biotechnology Information (NCBI) under the BioProject number PRJNA788986; Accession numbers SRR17283828-SRR17283841, SAMN25232627- SAMN25232640 and SRX14428743- SRX14428756.
