# Supplementary material for: Impact of meltwater flow intensity on the spatiotemporal heterogeneity of microbial mats in the McMurdo Dry Valleys, Antarctica
Source: ISME Commun. 2023 Jan 23;3:3. doi: 10.1038/s43705-022-00202-8 (PMC9870883; doi:10.1038/s43705-022-00202-8)
Supplement: Supplementary file 1 — Table S1 [file 43705_2022_202_MOESM1_ESM.pdf]

Table S1 Ash Free Dry Mass (AFDM), Chlorophyll a (Chl a) and AFDM to Chl a ratio values for green, orange, red and black mats collected from 1/9/17.

Color	AFDM	Chl a	AFDM:Chl a ratio
green	7.84	2.91	2.69
orange	14.3	3.06	4.69
red	22.8	2.52	9.06
black	27.8	0.229	121
