# Supplementary material for: Impact of meltwater flow intensity on the spatiotemporal heterogeneity of microbial mats in the McMurdo Dry Valleys, Antarctica
Source: ISME Commun. 2023 Jan 23;3:3. doi: 10.1038/s43705-022-00202-8 (PMC9870883; doi:10.1038/s43705-022-00202-8)
Supplement: Supplementary file 2 — Table S2 [file 43705_2022_202_MOESM2_ESM.pdf]

Table S2 Percent of reads mapping to assembled contigs.

Sample name	Input reads	Mapped reads	Percent mapped (%)
Black_11/30/16	21126758	15735076	74.48
Red_11/30/16	20784516	15479372	74.48
Black_12/18/16	11094418	5769906	52.01
Green_12/18/16	13588045	9308454	68.50
Orange_12/18/16	10469485	7505729	71.69
Red_12/18/16	12327150	7271758	58.99
Black_1/9/17	15623537	12174294	77.92
Green_1/9/17	14577043	10100914	69.29
Orange_1/9/17	21453836	16381587	76.36
Red_1/9/17	11584920	6875813	59.35
Black_1/26/17	13376586	8564903	64.03
Green_1/26/17	13987720	9480637	67.78
Orange_1/26/17	20033354	15415220	76.95
Red_1/26/17	5841525	2167901	37.11
