# Supplementary material for: Impact of meltwater flow intensity on the spatiotemporal heterogeneity of microbial mats in the McMurdo Dry Valleys, Antarctica
Source: ISME Commun. 2023 Jan 23;3:3. doi: 10.1038/s43705-022-00202-8 (PMC9870883; doi:10.1038/s43705-022-00202-8)
Supplement: Supplementary file 3 — Figure S1 [file 43705_2022_202_MOESM3_ESM.pdf]

Supplementary Figures

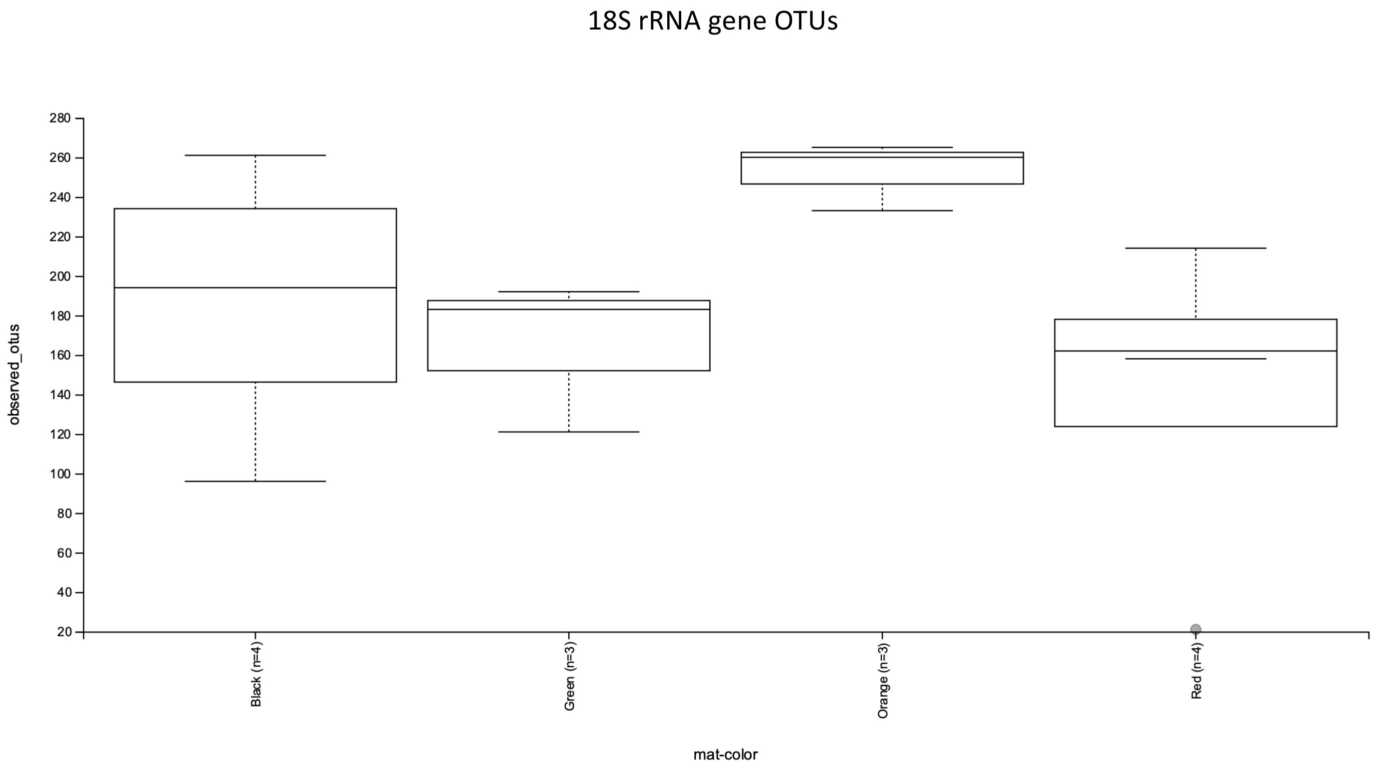

Figure S1 Boxplot of observed richness of 18S rRNA OTUs for mat samples grouped by black, green, red and orange mat types.
