# Supplementary material for: Impact of meltwater flow intensity on the spatiotemporal heterogeneity of microbial mats in the McMurdo Dry Valleys, Antarctica
Source: ISME Commun. 2023 Jan 23;3:3. doi: 10.1038/s43705-022-00202-8 (PMC9870883; doi:10.1038/s43705-022-00202-8)
Supplement: Supplementary file 4 — Figure S2 [file 43705_2022_202_MOESM4_ESM.pdf]

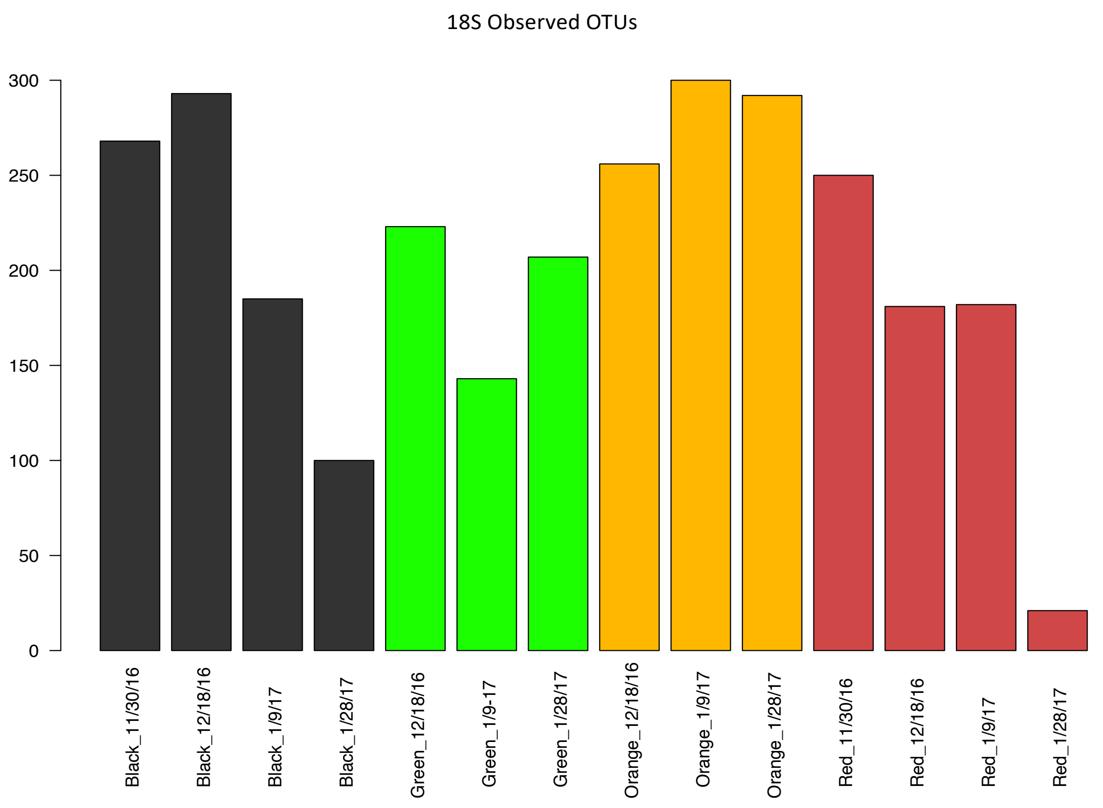

Figure S2 Bar graph of observed richness of 18S rRNA OTUs for black, green, red and orange mat individual samples.
