# Supplementary material for: Impact of meltwater flow intensity on the spatiotemporal heterogeneity of microbial mats in the McMurdo Dry Valleys, Antarctica
Source: ISME Commun. 2023 Jan 23;3:3. doi: 10.1038/s43705-022-00202-8 (PMC9870883; doi:10.1038/s43705-022-00202-8)
Supplement: Supplementary file 7 — Figure S5 [file 43705_2022_202_MOESM7_ESM.pdf]

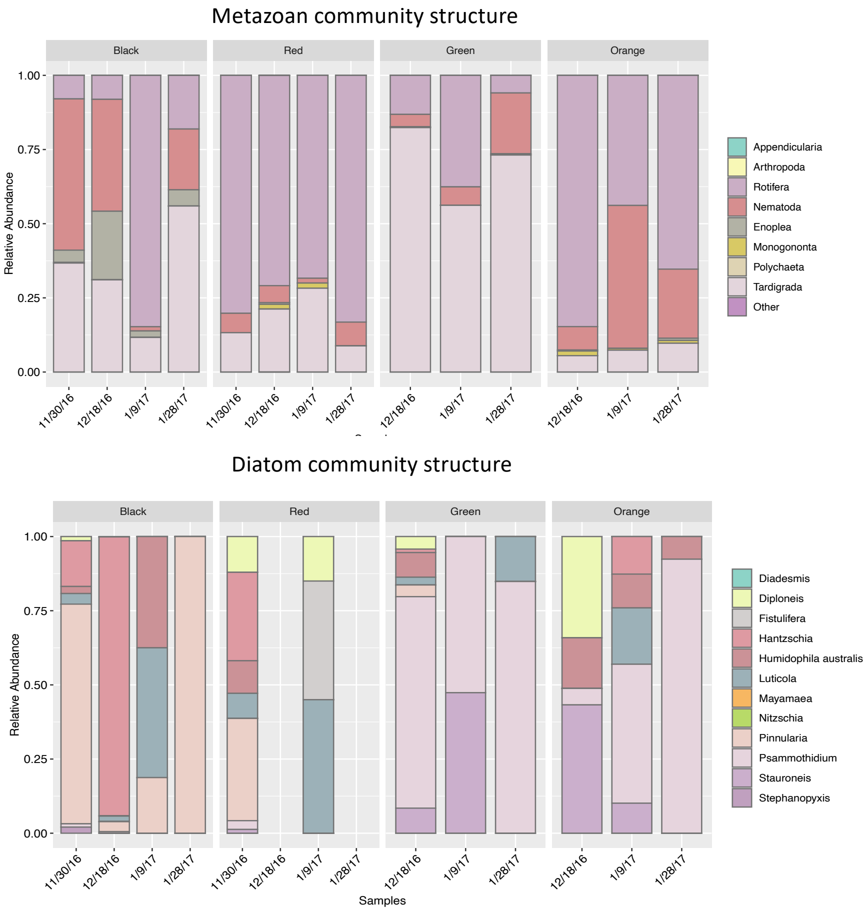

Figure S5 Relative abundance of (**top**) metazoan and (**bottom**) diatom community members derived from 18S rRNA ASVs. Diatoms were not detected in 12/18/16 or 1/28/17 red mat samples.
