# Supplementary material for: Impact of meltwater flow intensity on the spatiotemporal heterogeneity of microbial mats in the McMurdo Dry Valleys, Antarctica
Source: ISME Commun. 2023 Jan 23;3:3. doi: 10.1038/s43705-022-00202-8 (PMC9870883; doi:10.1038/s43705-022-00202-8)
Supplement: Supplementary file 8 — Figure S6 [file 43705_2022_202_MOESM8_ESM.pdf]

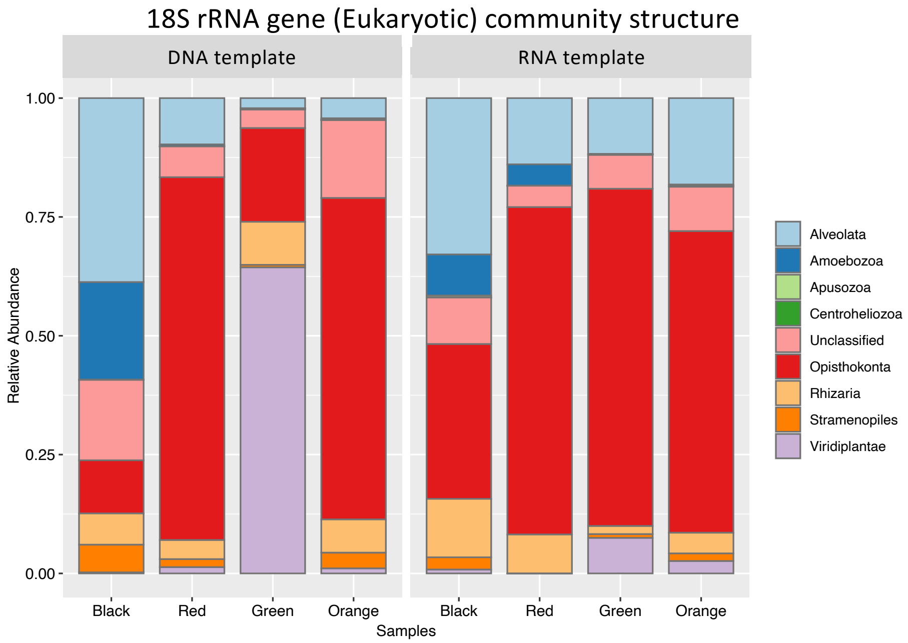

Figure S6 Comparison of eukaryote relative abundances from 01/28/17 black, red, green and orange mats showing differences between 18 rRNA communities sequenced from DNA (**left**) vs RNA (**right**) templates.
