# Supplementary material for: Impact of meltwater flow intensity on the spatiotemporal heterogeneity of microbial mats in the McMurdo Dry Valleys, Antarctica
Source: ISME Commun. 2023 Jan 23;3:3. doi: 10.1038/s43705-022-00202-8 (PMC9870883; doi:10.1038/s43705-022-00202-8)
Supplement: Supplementary file 9 — Figure S7 [file 43705_2022_202_MOESM9_ESM.pdf]

Black mat antifreeze activity assay: Dendritic burst
(stills per 0.2 seconds) Cooling rate: 0.074 °C per minute

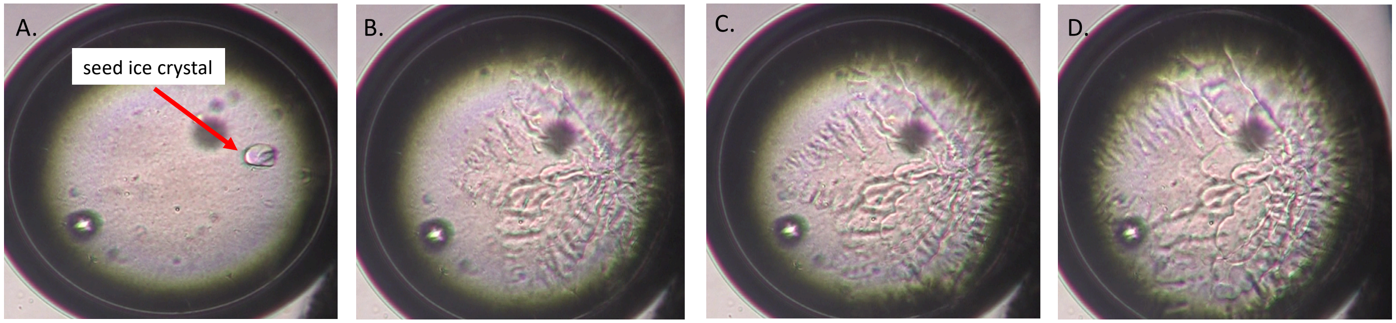

Control 1000 mOmol standard
(stills per 1 second) Cooling rate: 0.074 °C per minute

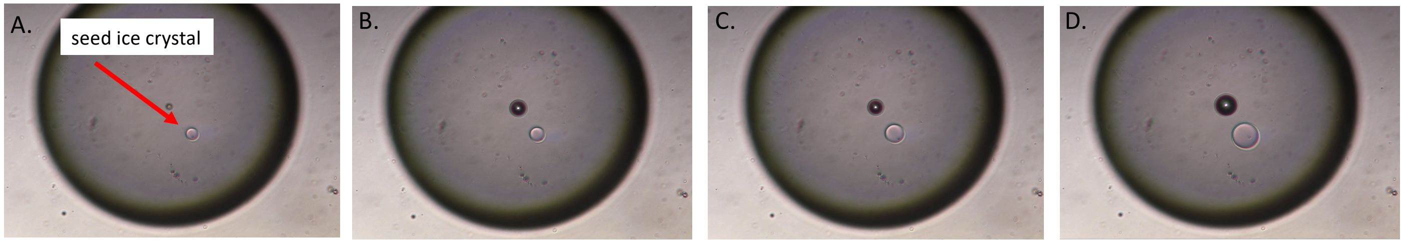

Figure S7 (top) Visualization of a dendritic burst of a seed ice crystal cooled at a rate of 0.074°C/min in a well with black mat sample lysate. Images are taken sequentially (**A**) 0.2 sec (**B**) 0.4 sec (**C**) 0.6 sec (**D**) 0.8 sec.
(**bottom**) A seed ice crystal cooled at a rate of 0.074°C/min in a well with 1000 mOsmol standard. Images are taken sequentially (**A**) 1 sec (**B**) 2 sec (**C**) 3 sec (**D**) 4 sec
