# Supplementary material for: Impact of meltwater flow intensity on the spatiotemporal heterogeneity of microbial mats in the McMurdo Dry Valleys, Antarctica
Source: ISME Commun. 2023 Jan 23;3:3. doi: 10.1038/s43705-022-00202-8 (PMC9870883; doi:10.1038/s43705-022-00202-8)
Supplement: Supplementary file 12 — Supporting Methods [file 43705_2022_202_MOESM12_ESM.pdf]

Supporting Methods

16S and 18S amplification, cDNA synthesis and sequencing protocol

A.) RNA and DNA were extracted from samples in accordance with NucleoMag RNA (Macherey-Nagel) and NucleoMag Plant (Macherey-Nagel) kit protocols

B.) 1-10 ng of total RNA was used to generate cDNA using the Life Technologies. SuperScript III First Strand Synthesis system with random hexamer primers.

C.) 4 µl of each sample template was used in a 25 µl PCR reaction, which contained, and a final primer concentration of 200 nM.

D.) Primers:

   a. 16S V4-V5 region
      515F-Y (5′-GTGYCAGCMGCCGCGGTAA) and
      926R (5′-CCGYCAATTYMTTTRAGTTT) (Parada et al. 2015)
   b. 18S V4 region
      F) CCAGCASCYGCGGTAATTCC
      R) ACTTTCGTTCTTGATYRA modified from (Stoeck et al. 2010)

E.) PCR Reaction:
   a. 5x buffer: 5ul
   b. TruFi Taq DNA Polymerase: 0.25ul
   c. Primer Mix: 1ul
   d. Templet: 4ul
   e. H20: 9.75

F.) A no template negative control for cDNA synthesis was used as a negative control for subsequent PCRs reactions.

G.) Thermocycling conditions included an initial denaturation at 95°C for 1 minutes, 30 cycles of 95°C for 15 seconds, 56°C for 15 seconds, 72°C for 30 minutes

H.) Reactions were cleaned using Ampure XP beads (Beckman Coulter, Brea CA). The final products were resuspended in 45 µL of elution buffer

I.) 1 µL was used to quantify the final product using' PicoGreen Quant-IT assay (thermoFihser) 20 ng and 30 ng of each 16S or 18S amplicon respectively were pooled separately for sequencing.

J.) 1-2 ul were used for final quantification on an Agilent TapeStation.

Metatranscriptomics amplification, cDNA synthesis and sequencing protocol

A.) 150ng of total RNA as input, ribosomal RNA was removed using Ribo-Zero Magnetic kits (Illumina).

B.)The rRNA-deplete total RNA was used for cDNA synthesis by Ovation RNA-Seq System V2 (TECAN, Redwood City, USA).

C.) Double stranded cDNA was fragmented using Covaries E210 system with the target size of 400bp.

D.) 100ng of fragmented cDNA as input into the Ovation Ultralow System V2 (TECAN, Redwood City, USA), following the manufactures protocol.

E.) Ampure XP beads (Beckman Coulter) were used for final library purification.

F.) Library quality was analyzed on a 2200 TapeStation System with Agilent High Sensitivity DNA 1000 ScreenTape System (Agilent Technologies, Santa Clara, CA, USA).
